# The Role of Histone Protein Modifications and Mutations in Histone Modifiers in Pediatric B-Cell Progenitor Acute Lymphoblastic Leukemia

**DOI:** 10.3390/cancers9010002

**Published:** 2017-01-03

**Authors:** Szymon Janczar, Karolina Janczar, Agata Pastorczak, Hani Harb, Adam J. W. Paige, Beata Zalewska-Szewczyk, Marian Danilewicz, Wojciech Mlynarski

**Affiliations:** 1 Department of Pediatrics, Oncology, Hematology and Diabetology, Medical University of Lodz, Lodz 91-738, Poland; agata.pastorczak@umed.lodz.pl (A.P.); beata.zalewska-szewczyk@umed.lodz.pl (B.Z.-S.); wojciech.mlynarski@umed.lodz.pl (W.M.); 2 Department of Pathology, Medical University of Lodz, Lodz 92-213, Poland; karolina.janczar@umed.lodz.pl (K.J.); marian.danilewicz@umed.lodz.pl (M.D.); 3 Institute of Laboratory Medicine and Pathobiochemistry, Molecular Diagnostics, Philipps University Marburg, Marburg 35043, Germany; harbh@staff.uni-marburg.de; 4 Department of Life Sciences, University of Bedfordshire, Bedfordshire LU1 3JU, UK; Adam.Paige@beds.ac.uk

**Keywords:** acute lymphoblastic leukemia, B lymphocytes, histone modifications, chromatin modifiers

## Abstract

While cancer has been long recognized as a disease of the genome, the importance of epigenetic mechanisms in neoplasia was acknowledged more recently. The most active epigenetic marks are DNA methylation and histone protein modifications and they are involved in basic biological phenomena in every cell. Their role in tumorigenesis is stressed by recent unbiased large-scale studies providing evidence that several epigenetic modifiers are recurrently mutated or frequently dysregulated in multiple cancers. The interest in epigenetic marks is especially due to the fact that they are potentially reversible and thus druggable. In B-cell progenitor acute lymphoblastic leukemia (BCP-ALL) there is a relative paucity of reports on the role of histone protein modifications (acetylation, methylation, phosphorylation) as compared to acute myeloid leukemia, T-cell ALL, or other hematologic cancers, and in this setting chromatin modifications are relatively less well studied and reviewed than DNA methylation. In this paper, we discuss the biomarker associations and evidence for a driver role of dysregulated global and loci-specific histone marks, as well as mutations in epigenetic modifiers in BCP-ALL. Examples of chromatin modifiers recurrently mutated/disrupted in BCP-ALL and associated with disease outcomes include *MLL1*, *CREBBP*, *NSD2*, and *SETD2*. Altered histone marks and histone modifiers and readers may play a particular role in disease chemoresistance and relapse. We also suggest that epigenetic regulation of B-cell differentiation may have parallel roles in leukemogenesis.

## 1. Introduction

### 1.1. Acute Lymphoblastic Leukemia in Children

Acute lymphoblastic leukemia (ALL) is the most common malignancy of childhood, and the majority of cases are classified as B-cell progenitor acute lymphoblastic leukemia (BCP-ALL). With current therapeutics, the cure rates exceed 80% but the treatment of relapsed or drug-resistant disease, and some molecular subtypes, remains challenging. With this high survival, there is little room for further improvement of outcomes based on escalation of the treatment intensity without unacceptable toxicity. Rather, the current effort is aimed at proper patient stratification and defining targetable genetic lesions that would allow for personalized therapy [1,2,3,4,5,6,7]. Here, we discuss the data on altered histone marks in B-cell progenitor acute lymphoblastic leukemia in children. Studying histone marks may not only help to understand BCP-ALL pathobiology but could identify prognostic biomarkers or provide rationale for novel therapeutic strategies. There are several agents, including FDA-approved drugs, aimed at dysregulated epigenetic states at different stages of development, and these can potentially enhance current therapeutic programs, as shown in preclinical models [8,9,10,11]. The aim of this review is to promote research into chromatin modification in BCP-ALL to facilitate future therapeutic interventions. It is not clear whether the apparent discrepancy between ALL, with relatively few data on histone modifiers, and acute myeloid leukemia (AML), in which most patients are reported to have mutations in epigenetic modifiers including in particular *DNMT3A* (DNA methyltransferase 3A), *TET2* methylcytosine hydroxylase (Ten-Elevan-Translocation-2) or *MLL1* (mixed lineage leukemia 1), is truly related to disease biology or a result of study bias [12,13,14]. The fact that a large number of studies used cytosine methylation profiling to classify BCP-ALL with prognostic significance is likely related to the relative ease of DNA methylation studying as compared to histone modifications [15,16,17,18,19,20,21,22,23,24]. There also appear to be more reports on the role of histone modifications and histone-modifying or chromatin-readers genes in T-cell ALL (with prominent reports on *DNMT3A*, *TET1*, *EZH2* (enhancer of zeste 2 polycomb repressive complex 2), *SUZ12* (SUZ12 polycomb repressive complex 2 subunit), *MLL2, SETD2* (SET domain containing 2), *PHF6* (PHD finger protein 6) and *BRD4* (bromodomain containing 4)), despite its relatively low frequency, than in BCP-ALL, and there is little overlap between the T-cell ALL and BCP-ALL data [25,26,27,28,29,30].

### 1.2. Histone Modifications

The traditional view of histones was that they are highly conserved proteins that provide the packaging of our genome. Now it is recognized that histone proteins have crucial roles in the interaction between effector proteins and DNA, and are themselves regulated by a number of modifications imposed by specialized sets of proteins creating an intricate interplay [31,32,33]. Some of these signatures appear relatively stable, others appear highly dynamic or might be subject to microenvironmental metabolic influences [32,33,34,35]. The number of recognized posttranslational, covalent histone protein modification is constantly growing. It is now apparent that they are involved in all basic cellular phenomena and in particular gene expression regulation, replication, and DNA repair [33,34]. Despite the introduction of high-throughput, genome-wide profiling methods combining chromatin immunoprecipitation with next-generation sequencing (ChIP-Seq) the data on the significance of such newly recognized marks in human diseases lag behind biochemical data from model organisms, though this is partly due to the relatively large quantities of input material required. The global and loci-specific level of each histone modification is the net result of the action of enzymes that can introduce the particular covalent modification (“writers”) or remove the mark (“erasers”), some of which also have non-histone targets. Apart from genomic location, the biological consequences of the histone marks are related to the action of proteins that interact with modified histones (“readers”) or recruit further molecules. The term “histone crosstalk” relates to the combinatorial, interdependent, and context-dependent effects of various histone modifications on the state and interpretation of other histone modifications [31,32,33,34,36,37,38]. While it was long known that several cancers are associated with dysregulated levels of several histone modifications, more recent, unbiased genome-wide studies reported that genes encoding chromatin modifiers and readers are among the most frequently mutated genes in cancer, providing strong evidence for their role in tumorigenesis [9,39,40,41,42,43,44,45].

In this review, we focus on three important and extensively described histone protein modifications: histone lysine acetylation, histone lysine methylation, and histone phosphorylation. For each of these major marks we discuss, if available in the literature, the correlative data related to their global or loci-specific levels; important data from pre-clinical models; and evidence of dysregulation of their writers, erasers, and readers in BCP-ALL. The data on other chromatin marks in BCP-ALL are very scarce as are data on their combinatorial effects (histone crosstalk).

### 1.3. Histone Lysine Acetylation

The *N*-acetylation of lysine residues in histone proteins may be considered the most important, or at least most extensively documented, of all histone marks. Histone acetylation is involved in gene transcription, chromatin structure, and DNA repair, which are basic cellular phenomena in physiology and in cancer [33,34,43,46]. Histone acetylation is the net result of the activities of histone lysine acetyltransferases (KATs) and histone deacetylases (HDACs) [33,36,38].

#### 1.3.1. Mutations/Rearrangements in Genes Involved in Histone Lysine Acetylation

CREBBP is a histone acetyltransferase that can acetylate various residues in several histones, and in particular H3K18 [33,38]. Recent unbiased, large-scale studies identify recurrent mutations in *CREBBP* in multiple cancers including bladder [42], salivary gland [47], esophageal [41], small-cell and non-small cell lung cancers [39,48], medulloblastoma [45], and lymphoid neoplasia [49,50,51,52,53,54,55,56,57,58,59,60,61,62]. In the setting of BCP-ALL *CREBBP* mutations (within the Histone acetyltransferases (HAT) domain) or deletions were shown to be very common in relapsed cases (18.3% of patients). These lesions were both acquired at relapse or already present at diagnosis, sometimes in subclones, suggesting a role in resistance to chemotherapy. Functional experiments suggested this is due to loss of HAT activity and transcriptional dysregulation [50]. Similar frequency of *CREBBP* gene mutations in relapsed cases was reported in a study by Mar et al. [60], and further studies demonstrated that *CREBBP* mutation are particularly prevalent in high hyperdiploid ALL [55,57,59]. However, despite the genetic evidence, we are far from understanding the role of CREBBP in tumorigenesis considering relatively low incidence of lymphoid neoplasia in Rubinstein-Taybi syndrome that is caused by *CREBBP* gene germline defects, and animal models of *CREBBP* loss that demonstrate hematologic abnormalities but not leukemia [63].

The role of *CREBBP* homolog, *EP300*, is less well established [50] however *EP300-ZNF384* was recently reported as a recurrent gene fusion in BCP-ALL [64].

The role of *CREBBP* and *EP300* in BCP-ALL and in lymphoid neoplasia is further complicated by the fact that they both contain bromodomains that recognize and bind to acetylated histones, thus also functioning as chromatin “readers” and recruiting other proteins to chromatin [65]. Inhibitors of CREBBP/EP300 bromodomains show promising pre-clinical activity in models of leukemia characterized by the presence of translocations leading to the presence of fusion proteins containing such bromodomains [66,67]. Several inhibitors of other bromodomains also showed therapeutic activity in BCP-ALL preclinical models, however it is difficult to conclude which mechanisms, and in particular which bromodomain-chromatin interactions and downstream effects, are critical for the effect. Nonetheless, these studies provide evidence for the potential of therapies targeting chromatin complexes in BCP-ALL [68,69]. Surprisingly, we identified no studies that examined the levels of *CREBBP* and other HATs expression in BCP-ALL.

#### 1.3.2. Differential Expression of Genes Involved in Histone Lysine Deacetylation

Several HDACs were demonstrated to be expressed in ALL at the higher levels than in normal bone marrow cells (HDAC2, -3, -6, -7, -8) and HDAC7 and -9 expression above median was associated with poor survival [70]. Another study reported overexpression of HDAC1, -2, -8 in ALL and that HDAC1, -2, 4, -11 expression is associated with unfavourable prognostic factors including poor prednisone response [71]. There is disappointingly little overlap between the studies, however it should be pointed out that both B- and T-cell ALL samples were included and their transcriptional profiles differ profoundly.

Important study by Sonnemann et al. [72] demonstrated that leukemic cells from ALL patients are characterized by increased histone deacetylase activity as compared to normal bone marrow cells using an enzymatic assay, which is a more direct and convincing proof of oncogenic hypoacetylation in cancer than association studies of HDACs expression. It would be interesting to see how this global HDAC activity level correlates with survival, or clinical/molecular data such as mutations in HAT genes, in a larger population of patients. In conclusion, the role for HDAC proteins in BCP-ALL is less well documented than that of some histone acetyltransferases. Various HDACs, Sir2 proteins (sirtuins) and histone acetyltransferases are differentially expressed in BCP-ALL molecular subtypes when publically available microarray datasets are analysed, data not shown. Despite a relative lack of clinicopathological data on their relevance, there are numerous in vitro and xenograft studies on the role of HDAC inhibitors in ALL, recently reviewed by Mummery et al. [73]. The most significant is the study of LBH589, a class I-II HDAC inhibitor, that demonstrated increased survival of human xenograft-implanted mice and synergy of LBH589 with vincristine and dexamethasone, accompanied by an increase in histone H3 and H4 acetylation in leukemic cells [74].

#### 1.3.3. The Association of Global and Loci-Specific Levels of Histone Acetylation

A rRecent study from our group confirmed and extended previous finding from adult ALL patients that associated loss of global Lys-4,-8,-12,-16-histone H4 acetylation with poor outcomes in pediatric BCP-ALL. Additionally, we demonstrated that in BCP-ALL relatively preserved level of histone H4 acetylation is linked with the presence of *ETV6-RUNX1* gene fusion, *PAX5* deletions, and deletions in genes related B-cell differentiation [75,76,77]. A study by Bachmann et al. [78] demonstrated that loss of histone H3 acetylation (H3K9Ac) at *BIM* locus is associated with glucocorticoid resistance in xenograft models and in primary patient samples.

### 1.4. Histone Lysine Methylation

Histone lysine methylation is another of the major histone marks. Methylation of various lysine residues of histone proteins is the net result of the activities of histone lysine methylases and demethylases. Histone lysine methylation is mainly associated with chromatin state and transcriptional regulation [10,33,38].

There are no reports on the role of histone arginine methylation in BCP-ALL.

#### Mutations/Rearrangements in Genes Involved in Histone Lysine Methylation

Several histone methyltransferases are implicated in BCP-ALL pathogenesis, including *MLL1*. Mixed lineage leukemia (*MLL1*, recently known as *KMT2A*) translocations are found in around 70% of infant leukemia and 5% of BCP-ALL. MLL1 is a member of SET domain-containing histone lysine methyltransferases that also contain EZH2, NSD1, and SET7/9, which are also frequently disrupted in hematologic neoplasia [79,80,81]. In BCP-ALL t(11q23) *MLL1* gene translocations (best described *MLL1-AF4*) are associated with high risk disease and are an established cytogenetic risk factor for a few decades [81,82]. *MLL1/KMT2A* is one of the most frequently mutated genes in cancer [83]. MLL1 is a H3K4 methyltransferase and this activity is dependent on its SET domain. H3K4 methylation is typically associated with transcriptional activation and euchromatin [33,38]. The role of MLL1 fusion oncoproteins in leukemogenesis is believed to be related to overexpression of target genes, such as *HOXA* homeobox gene cluster that are normally tightly regulated in hematopoietic progenitors, due to aberrant histone H3 methylation. However, it is not clear to what extent the role of MLL1 in leukemagenesis is related to its wild-type methyltransferase activity and what is the role of the particular fusion partners, of which more than 50 were characterized, especially that SET domain is frequently lost in resulting fusion proteins. It must be also noted that, similarly to CREBBP, MLL1 contains a bromodomain that provides a potential platform for interaction with various chromatin complexes [80,81]. This is further complicated by the fact that several MLL1 fusion partners interact with and recruit DOT1L methyltransferase, specific for H3K79 (which is also associated with transcriptional activation). Whatever the exact mechanism, *MLL1* lesions are believed to be associated with aberrant histone methylation and overexpression of target genes. Interestingly, more recent studies suggest that H3K79met patterns are more consistently associated with *MLL1*-rearranged leukemia than H3K4met profiles, and DOT1L is essential for MLL1-driven leukemogenesis as a member of MLL1-associated multiprotein complexes. Importantly, DOT1L inhibitors may be selectively active against *MLL*-rearranged cells (including both AF9 and AF4 fusion partners) [79,80,81,84,85,86,87,88]. Several other approaches, apart from DOT1L inhibition, are surveyed in the setting of *MLL*-rearranged leukemia, and another member of the multiprotein complex associated with MLL1 that could be targeted in ALL is WD repeat 5 (WDR5), however this was not studied in pediatric populations or disease models [89]. A therapeutic potential and antileukemic activity was recently demonstrated in a study of the MM-401 compound targeting MLL1/KMT2A H3K4 methyltransferase activity in mixed lineage leukemia, suggesting that wild type *MLL1* is necessary for MLL1-rearrangement driven leukemia [90]. As *MLL1*-driven leukemia is also associated with aberrant DNA methylation patterns, another suggested therapeutic strategy is using hypomethylating agents [81]. Similarly, HDAC inhibitors are also evaluated in this setting based on the assumption that histone methylation pattern is read in the context of other chromatin marks [81].

Other histone methyltransferases implicated in BCP-ALL leukemagenesis and this include *NSD2*, *SETD2*, and *EZH2*.

NSD2 (nuclear receptor-binding SET domain protein 2) histone lysine methyltransferase is responsible for mono- and dimethylation of H3K36. A specific E1099K mutation in *NSD2* gene increased H3K36 dimethylation in several ALL cell lines and xenograft studies. Further, the E1099K variant was shown to be a frequent feature of BCP-ALL with *ETV6-RUNX1* fusion gene [91]. The recurrent character of *NSD2* gene mutations in BCP-ALL was confirmed by Huether et al. [44] and Oyer et al. [92]. H3K36 is normally unmethylated, but it remains to be established how this gain in methylation contributes to leukemogenesis. It was suggested that it might be due to global transcriptional dysregulation caused by concomitant decrease in H3K27me3 [92].

SETD2 is an another H3K36 methyltransferase whose mutations are reported in BCP-ALL at a relatively high frequency (12% of the entire cohort). The frequency of *SETD2* gene mutations is increased in *MLL1*- and *ETV6-RUNX1* rearranged cases and is also increased at relapse [60]. In AML *SETD2* defects are associated with a global loss of H3K36 trimethylation, but this was not studied in BCP-ALL [93].

A recent study by Schafer et al. [94] found a relatively low frequency (1.3%) of mutation in another histone methyltransferase *EZH2* in ALL, similar to low *EZH2* mutation prevalence in ALL seen previously [50]. *EZH2* gene mutations might be enriched in hypodiploid ALL [95].

Histone lysine demethylases have been strongly implicated in T-cell acute lymphoblastic leukemia [30,96], but there is a lack of studies reporting on their alterations in BCP-ALL.

We identified no studies involving pediatric BCP-ALL patients documenting differential expression of genes involved in histone lysine methylation, the associations of global levels of histone methylation, or the associations of loci-specific patterns of histone methylation. However, interestingly, in an in vitro model, loss of IKAROS (IKZF1), that is one of the main drivers of high-risk leukemia and whose activity restoration is attempted in preclinical models, was associated with decreased level of global H3K4 methylation [97,98].

### 1.5. Histone Phosphorylation

Histone phosphorylation plays a major regulatory role in transcription, chromatin condensation, mitosis, apoptosis, and DNA replication. This is a highly dynamic chromatin modification controlled by several protein kinases and phosphatases [10,33,38].

#### Histone Phosphorylation in BCP-ALL

Aberrant phosphorylation of several histone proteins and mutations in genes encoding for proteins involved in histone phosphorylation are reported in multiple cancers [10]. There is a lack of such reports in the setting of acute lymphoblastic leukemia, which may represent true biological features of the disease or be related to the fact that ALL is relatively understudied. Still, it is highly likely that other histone marks are “read” in the context of phosphorylation of residues in the vicinity and the so far scarce data on histone phosphorylation in BCP-ALL are necessary to fully interpret other chromatin marks. The notable exception is Janus kinase (JAK2, JAK3), which is a site of recurrent rearrangements in ALL that are of biological and clinical significance [99,100]. JAK2 was recently reported to be able to phosphorylate histone H3 at tyrosine 41 (H3Y41), which leads to dissociation of some effector proteins from chromatin, and global H3Y41 levels are elevated in cell lines with constitutively active JAK2 [101]. To the best of our knowledge, this role of JAK2 was not studied in the setting of acute lymphoblastic leukemia. Importantly JAK proteins can be effectively targeted with ruxolitinib [100,102] and in preclinical models with HSP90 (heat shock protein 90 kDa) inhibitors [103]. Other than that, we are not aware of studies reporting on mutations/rearrangements or differential expression in genes involved in histone phosphorylation, nor on the associations of global levels or loci-specific levels of histone phosphorylation. Similarly, several further histone marks are known and were shown to play role in basic cellular phenomena but no data on their role in BCP-ALL exists.

### 1.6. Histone Gene Disruption in BCP-ALL

Histone genes themselves may be targets in tumorigenesis. Mullighan et al. [95] demonstrated 8.1% frequency of 6p22 deletions in a histone cluster region in BCP-ALL. This deletion may be enriched in cases with Down syndrome and hypodiploidy. Loudin et al. [104] reported deletion in a histone gene cluster at 6p22 in 22% of Down-syndrome ALL patients, and in 3.1% patients without Down syndrome, and gene expression analysis confirmed lower expression of several histone genes in cases with homozygous deletions. The same study reports a few likely functional histone genes mutations in the Down-syndrome patients without those 6p22 deletions. More recently, Holmfeldt [105] reported a relatively high frequency of histone cluster deletion (19.1%) in hypodiploid ALL. There are no data on the prognostic significance of these alterations.

### 1.7. The Evidence for the Role of Histone Modification in B-Cell Differentiation

Another reason to expect a role for histone marks in BCP-ALL leukemagenesis is their involvement in normal B-cell development, as corrupted developmental regulation frequently plays a role in cancer, including leukemia [106,107,108,109]. Apart from the fact that various epigenetic events likely regulate all stages of B-cell progenitor development [110], in the context of B-cell differentiation histone marks were particularly implicated in the regulation of V(D)J rearrangement at immunoglobulin loci. It was demonstrated that RAG (recombination-activating genes) recombinases specifically recognize H3K4me3, and loss of this recognition in laboratory models or in patients with immunodeficiency is related to severely impaired V(D)J recombination [111,112,113,114,115]. The activity and localization of recombination complexes is also regulated by histone acetylation [116,117,118]. Aberrant RAG recombinase activity was recently strongly implicated in BCP-ALL leukemogenesis in several reports [119,120,121] and it might be expected that aberrant histone methylation and acetylation marks play a role in illegitimate or excessive recombination.

## 2. Conclusions

**Figure 1:**
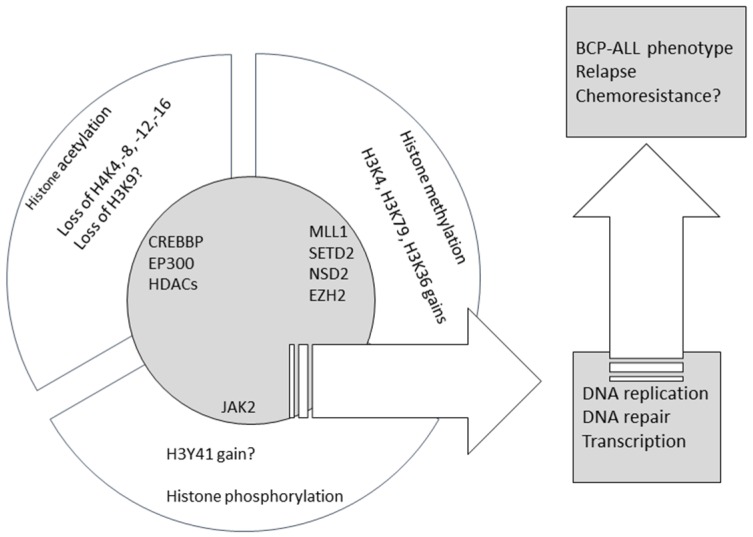
The summary of the published data on aberrant histone marks and mutations in chromatin modifiers in BCP-ALL.

Recent large unbiased studies provided strong evidence for the role of factors involved in histone modifications in B-cell progenitor leukemogenesis, and their association with chemoresistance and relapse. Table 1 summarizes the evidence on the recurrent character of the lesions in genes encoding histone mark writers and erasers, whereas Figure 1 summarizes the data presented here on aberrant histone marks and related mutations in chromatin modifiers. Still, the area appears relatively understudied when compared to other cancers, including hematologic neoplasia. This gap must be filled to enable novel and targeted therapeutic interventions. Drugs targeting histone modifiers and readers are entering clinical trials (completed or active clinical trials of such agents in pediatric BCP-ALL are presented in Table 2), and are expected to play a role in multidrug regimens combined with ‘traditional’ cytotoxics. Their use may prove beneficial in unselected BCP-ALL cases irrespective of molecular build-up but we hope that their future use will be guided by data on genomic and epigenomic lesions in a particular patient to achieve maximal clinical benefit with minimal toxicities.

## Figures and Tables

**Table 1:** Mutations/rearrangements of histone writers and erasers in B-cell progenitor acute lymphoblastic leukemia (BCP-ALL).

Gene (Reference)	Histone-Modifying Function	Frequency of Mutations/Rearrangements in BCP-ALL	Subgroups Enriched
*CREBBP* [50,51,53,55,57]	H3K18 acetyltransferase (and other H3/H4 residues)	Rare in cases without hyperdiploidy and relapse	Relapse, hyperdiploidy
*EP300* [50,55]	H3K18 acetyltransferase (and other H3/H4 residues)	<1%	-
*MLL1* [3,5,6,82,122,123]	H3K4 methyltransferase	5%	-
*NSD2* [44,91]	H3K36 methyltransferase	Not documented in unselected patients, up to 14% in subgroups	*ETV6-RUNX1-*rearranged
*SETD2* [44,60,93,124]	H3K36 methyltransferase	12%	*MLL*- and *ETV6-RUNX1-* rearrangements, relapse
*EZH2* [44,55,94]	H3K4 methyltransferase	1.3%	hypodiploidy
*JAK2* [99,100,125,126]	H3Y41 phosphorylase	Not determined in unselected patients, up to 10% in high risk disease	*BCR-ABL1-*like, Down syndrome, high-risk disease

**Table 2:** Active and completed clinical trials of drugs potentially targeting histone mark writers and erasers in pediatric BCP-ALL (as accessed on 25 October 2016, at www.clinicaltrails.gov).

Study Identifier	Start Year	Drug Targeting (or Potentially Targeting) Histone Modifications	ALL Population	Phase	Status
NCT00053963	2002	FR901228 (HDACi)	Refractory disease (0–21 years)	1	completed
NCT00217412	2005	Vorinostat (HDACi)	Relapsed or refractory disease (1–21 years)	1	completed
NCT00882206	2009	Vorinostat (HDACi)	Relapsed or refractory disease (2–60 years)	2	completed
NCT01251965	2010	Ruxolitinib (JAK1/JAK2 inhibitor)	Relapsed or refractory disease (14 years or older)	1/2	completed
NCT01321346	2011	Panobinostat (HDACi)	Refractory disease (8–21 years)	1	completed
NCT02141828	2014	EPZ-5676 (DOT1L blocker)	Relapsed or refractory disease (0–18 years) MLL-rearranged	1	completed
NCT02419755	2015	Vorinostat (HDACi)	Relapsed or refractory disease (0–21 years) MLL-rearranged	2	recruiting
NCT02420717	2015	Ruxolitinib (JAK1/JAK2 inhibitor)	Ph-like (10 years or older)	2	recruiting
NCT02723994	2016	Ruxolitinib (JAK1/JAK2 inhibitor)	CRLF2-rearranged and/or JAK Pathway-mutant (1–21 years)	2	recruiting

## References

[1] Hunger S.P., Raetz E.A., Loh M.L., Mullighan C.G. (2011). Improving outcomes for high-risk ALL: Translating new discoveries into clinical care. Pediatr. Blood Cancer.

[2] Moorman A.V., Enshaei A., Schwab C., Wade R., Chilton L., Elliott A., Richardson S., Hancock J., Kinsey S.E., Mitchell C.D. (2014). A novel integrated cytogenetic and genomic classification refines risk stratification in pediatric acute lymphoblastic leukemia. Blood.

[3] Mullighan C.G. (2009). Genomic analysis of acute leukemia. Int. J. Lab. Hematol..

[4] Mullighan C.G., Downing J.R. (2009). Global genomic characterization of acute lymphoblastic leukemia. Semin. Hematol..

[5] Mullighan C.G. (2011). Genomic profiling of B-progenitor acute lymphoblastic leukemia. Best Pract. Res. Clin. Haematol..

[6] Mullighan C.G. (2011). New strategies in acute lymphoblastic leukemia: Translating advances in genomics into clinical practice. Clin. Cancer Res..

[7] Pui C.H., Mullighan C.G., Evans W.E., Relling M.V. (2012). Pediatric acute lymphoblastic leukemia: Where are we going and how do we get there?. Blood.

[8] Bolden J.E., Peart M.J., Johnstone R.W. (2006). Anticancer activities of histone deacetylase inhibitors. Nat. Rev. Drug Discov..

[9] Chi P., Allis C.D., Wang G.G. (2010). Covalent histone modifications--miswritten, misinterpreted and mis-erased in human cancers. Nat. Rev. Cancer.

[10] Dawson M.A., Kouzarides T. (2012). Cancer epigenetics: From mechanism to therapy. Cell.

[11] Dawson M.A., Kouzarides T., Huntly B.J. (2012). Targeting epigenetic readers in cancer. N. Engl. J. Med..

[12] Wouters B.J., Delwel R. (2016). Epigenetics and approaches to targeted epigenetic therapy in acute myeloid leukemia. Blood.

[13] Plass C., Oakes C., Blum W., Marcucci G. (2008). Epigenetics in acute myeloid leukemia. Semin. Oncol..

[14] Pastore F., Levine R.L. (2016). Epigenetic regulators and their impact on therapy in acute myeloid leukemia. Haematologica.

[15] Burke M.J., Bhatla T. (2014). Epigenetic modifications in pediatric acute lymphoblastic leukemia. Front. Pediatr..

[16] Busche S., Ge B., Vidal R., Spinella J.F., Saillour V., Richer C., Healy J., Chen S.H., Droit A., Sinnett D., Pastinen T. (2013). Integration of high-resolution methylome and transcriptome analyses to dissect epigenomic changes in childhood acute lymphoblastic leukemia. Cancer Res..

[17] Chatterton Z., Morenos L., Saffery R., Craig J.M., Ashley D., Wong N.C. (2010). DNA methylation and miRNA expression profiling in childhood B-cell acute lymphoblastic leukemia. Epigenomics.

[18] Chatterton Z., Morenos L., Mechinaud F., Ashley D.M., Craig J.M., Sexton-Oates A., Halemba M.S., Parkinson-Bates M., Ng J., Morrison D. (2014). Epigenetic deregulation in pediatric acute lymphoblastic leukemia. Epigenetics.

[19] Cimmino L., Aifantis I. (2012). Fingerprinting acute leukemia: DNA methylation profiling of B-acute lymphoblastic leukemia. Cancer Discov..

[20] Davidsson J., Lilljebjörn H., Andersson A., Veerla S., Heldrup J., Behrendtz M., Fioretos T., Johansson B. (2009). The DNA methylome of pediatric acute lymphoblastic leukemia. Hum. Mol. Genet..

[21] Milani L., Lundmark A., Kiialainen A., Nordlund J., Flaegstad T., Forestier E., Heyman M., Jonmundsson G., Kanerva J., Schmiegelow K. (2010). DNA methylation for subtype classification and prediction of treatment outcome in patients with childhood acute lymphoblastic leukemia. Blood.

[22] Nordlund J., Bäcklin C.L., Wahlberg P., Busche S., Berglund E.C., Eloranta M.L., Flaegstad T., Forestier E., Frost B.M., Harila-Saari A. (2013). Genome-wide signatures of differential DNA methylation in pediatric acute lymphoblastic leukemia. Genome Biol..

[23] Nordlund J., Bäcklin C.L., Zachariadis V., Cavelier L., Dahlberg J., Öfverholm I., Barbany G., Nordgren A., Övernäs E., Abrahamsson J. (2015). DNA methylation-based subtype prediction for pediatric acute lymphoblastic leukemia. Clin. Epigenet..

[24] Sandoval J., Heyn H., Méndez-González J., Gomez A., Moran S., Baiget M., Melo M., Badell I., Nomdedéu J.F., Esteller M. (2013). Genome-wide DNA methylation profiling predicts relapse in childhood B-cell acute lymphoblastic leukaemia. Br. J. Haematol..

[25] Peirs S., Van der Meulen J., Van de Walle I., Taghon T., Speleman F., Poppe B., Van Vlierberghe P. (2015). Epigenetics in T-cell acute lymphoblastic leukemia. Immunol. Rev..

[26] Fujikawa D., Nakagawa S., Hori M., Kurokawa N., Soejima A., Nakano K., Yamochi T., Nakashima M., Kobayashi S., Tanaka Y. (2016). Polycomb-dependent epigenetic landscape in adult T-cell leukemia. Blood.

[27] Knoechel B., Roderick J.E., Williamson K.E., Zhu J., Lohr J.G., Cotton M.J., Gillespie S.M., Fernandez D., Ku M., Wang H. (2014). An epigenetic mechanism of resistance to targeted therapy in T cell acute lymphoblastic leukemia. Nat. Genet..

[28] Benyoucef A., Palii C.G., Wang C., Porter C.J., Chu A., Dai F., Tremblay V., Rakopoulos P., Singh K., Huang S. (2016). UTX inhibition as selective epigenetic therapy against TAL1-driven T-cell acute lymphoblastic leukemia. Genes Dev..

[29] Bhatla T., Wang J., Morrison D.J., Raetz E.A., Burke M.J., Brown P., Carroll W.L. (2012). Epigenetic reprogramming reverses the relapse-specific gene expression signature and restores chemosensitivity in childhood B-lymphoblastic leukemia. Blood.

[30] Ntziachristos P., Tsirigos A., Welstead G.G., Trimarchi T., Bakogianni S., Xu L., Loizou E., Holmfeldt L., Strikoudis A., King B. (2014). Contrasting roles of histone 3 lysine 27 demethylases in acute lymphoblastic leukaemia. Nature.

[31] Jenuwein T., Allis C.D. (2001). Translating the histone code. Science.

[32] Tessarz P., Kouzarides T. (2014). Histone core modifications regulating nucleosome structure and dynamics. Nat. Rev. Mol. Cell Biol..

[33] Kouzarides T. (2007). Chromatin modifications and their function. Cell.

[34] Bannister A.J., Kouzarides T. (2011). Regulation of chromatin by histone modifications. Cell Res..

[35] Lee J.V., Carrer A., Shah S., Snyder N.W., Wei S., Venneti S., Worth A.J., Yuan Z.F., Lim H.W., Liu S. (2014). Akt-dependent metabolic reprogramming regulates tumor cell histone acetylation. Cell Metab..

[36] Allis C.D., Berger S.L., Cote J., Dent S., Jenuwien T., Kouzarides T., Pillus L., Reinberg D., Shi Y., Shiekhattar R. (2007). New nomenclature for chromatin-modifying enzymes. Cell.

[37] Bartke T., Kouzarides T. (2011). Decoding the chromatin modification landscape. Cell Cycle.

[38] Kouzarides T. (2007). SnapShot: Histone-modifying enzymes. Cell.

[39] Campbell J.D., Alexandrov A., Kim J., Wala J., Berger A.H., Pedamallu C.S., Shukla S.A., Guo G., Brooks A.N., Murray B.A. (2016). Distinct patterns of somatic genome alterations in lung adenocarcinomas and squamous cell carcinomas. Nat. Genet..

[40] Fraga M.F., Ballestar E., Villar-Garea A., Boix-Chornet M., Espada J., Schotta G., Bonaldi T., Haydon C., Ropero S., Petrie K. (2005). Loss of acetylation at Lys16 and trimethylation at Lys20 of histone H4 is a common hallmark of human cancer. Nat. Genet..

[41] Gao Y.B., Chen Z.L., Li J.G., Hu X.D., Shi X.J., Sun Z.M., Zhang F., Zhao Z.R., Li Z.T., Liu Z.Y. (2014). Genetic landscape of esophageal squamous cell carcinoma. Nat. Genet..

[42] Gui Y., Guo G., Huang Y., Hu X., Tang A., Gao S., Wu R., Chen C., Li X., Zhou L. (2011). Frequent mutations of chromatin remodeling genes in transitional cell carcinoma of the bladder. Nat. Genet..

[43] Ge Z., Nair D., Guan X., Rastogi N., Freitas M.A., Parthun M.R. (2013). Sites of acetylation on newly synthesized histone H4 are required for chromatin assembly and DNA damage response signaling. Mol. Cell. Biol..

[44] Huether R., Dong L., Chen X., Wu G., Parker M., Wei L., Ma J., Edmonson M.N., Hedlund E.K., Rusch M.C. (2014). The landscape of somatic mutations in epigenetic regulators across 1000 paediatric cancer genomes. Nat. Commun..

[45] Robinson G., Parker M., Kranenburg T.A., Lu C., Chen X., Ding L., Phoenix T.N., Hedlund E., Wei L., Zhu X. (2012). Novel mutations target distinct subgroups of medulloblastoma. Nature.

[46] Dang W., Steffen K.K., Perry R., Dorsey J.A., Johnson F.B., Shilatifard A., Kaeberlein M., Kennedy B.K., Berger S.L. (2009). Histone H4 lysine 16 acetylation regulates cellular lifespan. Nature.

[47] Ho A.S., Kannan K., Roy D.M., Morris L.G., Ganly I., Katabi N., Ramaswami D., Walsh L.A., Eng S., Huse J.T. (2013). The mutational landscape of adenoid cystic carcinoma. Nat. Genet..

[48] Peifer M., Fernández-Cuesta L., Sos M.L., George J., Seidel D., Kasper L.H., Plenker D., Leenders F., Sun R., Zander T. (2012). Integrative genome analyses identify key somatic driver mutations of small-cell lung cancer. Nat. Genet..

[49] Lohr J.G., Stojanov P., Lawrence M.S., Auclair D., Chapuy B., Sougnez C., Cruz-Gordillo P., Knoechel B., Asmann Y.W., Slager S.L. (2012). Discovery and prioritization of somatic mutations in diffuse large B-cell lymphoma (DLBCL) by whole-exome sequencing. Proc. Natl. Acad. Sci. USA.

[50] Mullighan C.G., Zhang J., Kasper L.H., Lerach S., Payne-Turner D., Phillips L.A., Heatley S.L., Holmfeldt L., Collins-Underwood J.R., Ma J. (2011). CREBBP mutations in relapsed acute lymphoblastic leukaemia. Nature.

[51] Mullighan C.G. (2014). The genomic landscape of acute lymphoblastic leukemia in children and young adults. Hematol. Am. Soc. Hematol. Educ. Program.

[52] Pasqualucci L., Dominguez-Sola D., Chiarenza A., Fabbri G., Grunn A., Trifonov V., Kasper L.H., Lerach S., Tang H., Ma J. (2011). Inactivating mutations of acetyltransferase genes in B-cell lymphoma. Nature.

[53] Zhang J., Mullighan C.G., Harvey R.C., Wu G., Chen X., Edmonson M., Buetow K.H., Carroll W.L., Chen I.M., Devidas M. (2011). Key pathways are frequently mutated in high-risk childhood acute lymphoblastic leukemia: A report from the Children’s Oncology Group. Blood.

[54] Bouska A., Zhang W., Gong Q., Iqbal J., Scuto A., Vose J., Ludvigsen M., Fu K., Weisenburger D.D., Greiner T.C. (2016). Combined copy number and mutation analysis identifies oncogenic pathways associated with transformation of follicular lymphoma. Leukemia.

[55] Chen C., Bartenhagen C., Gombert M., Okpanyi V., Binder V., Röttgers S., Bradtke J., Teigler-Schlegel A., Harbott J., Ginzel S. (2015). Next-generation-sequencing of recurrent childhood high hyperdiploid acute lymphoblastic leukemia reveals mutations typically associated with high risk patients. Leuk. Res..

[56] Da Silva Almeida A.C., Abate F., Khiabanian H., Martinez-Escala E., Guitart J., Tensen C.P., Vermeer M.H., Rabadan R., Ferrando A., Palomero T. (2015). The mutational landscape of cutaneous T cell lymphoma and Sézary syndrome. Nat. Genet..

[57] Inthal A., Zeitlhofer P., Zeginigg M., Morak M., Grausenburger R., Fronkova E., Fahrner B., Mann G., Haas O.A., Panzer-Grümayer R. (2012). CREBBP HAT domain mutations prevail in relapse cases of high hyperdiploid childhood acute lymphoblastic leukemia. Leukemia.

[58] Lunning M.A., Green M.R. (2015). Mutation of chromatin modifiers; an emerging hallmark of germinal center B-cell lymphomas. Blood Cancer J..

[59] Malinowska-Ozdowy K., Frech C., Schönegger A., Eckert C., Cazzaniga G., Stanulla M., zur Stadt U., Mecklenbräuker A., Schuster M., Kneidinger D. (2015). KRAS and CREBBP mutations: A relapse-linked malicious liaison in childhood high hyperdiploid acute lymphoblastic leukemia. Leukemia.

[60] Mar B.G., Bullinger L.B., McLean K.M., Grauman P.V., Harris M.H., Stevenson K., Neuberg D.S., Sinha A.U., Sallan S.E., Silverman L.B. (2014). Mutations in epigenetic regulators including SETD2 are gained during relapse in paediatric acute lymphoblastic leukaemia. Nat. Commun..

[61] Morin R.D., Mendez-Lago M., Mungall A.J., Goya R., Mungall K.L., Corbett R.D., Johnson N.A., Severson T.M., Chiu R., Field M. (2011). Frequent mutation of histone-modifying genes in non-Hodgkin lymphoma. Nature.

[62] Okosun J., Bödör C., Wang J., Araf S., Yang C.Y., Pan C., Boller S., Cittaro D., Bozek M., Iqbal S. (2014). Integrated genomic analysis identifies recurrent mutations and evolution patterns driving the initiation and progression of follicular lymphoma. Nat. Genet..

[63] Zimmer S.N., Zhou Q., Zhou T., Cheng Z., Abboud-Werner S.L., Horn D., Lecocke M., White R., Krivtsov A.V., Armstrong S.A. (2011). Crebbp haploinsufficiency in mice alters the bone marrow microenvironment, leading to loss of stem cells and excessive myelopoiesis. Blood.

[64] Gocho Y., Kiyokawa N., Ichikawa H., Nakabayashi K., Osumi T., Ishibashi T., Ueno H., Terada K., Oboki K., Sakamoto H. (2015). A novel recurrent EP300-ZNF384 gene fusion in B-cell precursor acute lymphoblastic leukemia. Leukemia.

[65] Josling G.A., Selvarajah S.A., Petter M., Duffy M.F. (2012). The role of bromodomain proteins in regulating gene expression. Genes.

[66] Ciceri P., Müller S., O’Mahony A., Fedorov O., Filippakopoulos P., Hunt J.P., Lasater E.A., Pallares G., Picaud S., Wells C. (2014). Dual kinase-bromodomain inhibitors for rationally designed polypharmacology. Nat. Chem. Biol..

[67] Picaud S., Fedorov O., Thanasopoulou A., Leonards K., Jones K., Meier J., Olzscha H., Monteiro O., Martin S., Philpott M. (2015). Generation of a Selective Small Molecule Inhibitor of the CBP/p300 Bromodomain for Leukemia Therapy. Cancer Res..

[68] Ott C.J., Kopp N., Bird L., Paranal R.M., Qi J., Bowman T., Rodig S.J., Kung A.L., Bradner J.E., Weinstock D.M. (2012). BET bromodomain inhibition targets both c-Myc and IL7R in high-risk acute lymphoblastic leukemia. Blood.

[69] Da Costa D., Agathanggelou A., Perry T., Weston V., Petermann E., Zlatanou A., Oldreive C., Wei W., Stewart G., Longman J., Smith E., Kearns P., Knapp S., Stankovic T. (2013). BET inhibition as a single or combined therapeutic approach in primary paediatric B-precursor acute lymphoblastic leukaemia. Blood Cancer J..

[70] Moreno D.A., Scrideli C.A., Cortez M.A., de Paula Queiroz R., Valera E.T., da Silva Silveira V., Yunes J.A., Brandalise S.R., Tone L.G. (2010). Differential expression of HDAC3, HDAC7 and HDAC9 is associated with prognosis and survival in childhood acute lymphoblastic leukaemia. Br. J. Haematol..

[71] Gruhn B., Naumann T., Gruner D., Walther M., Wittig S., Becker S., Beck J.F., Sonnemann J. (2013). The expression of histone deacetylase 4 is associated with prednisone poor-response in childhood acute lymphoblastic leukemia. Leuk. Res..

[72] Sonnemann J., Gruhn B., Wittig S., Becker S., Beck J.F. (2012). Increased activity of histone deacetylases in childhood acute lymphoblastic leukaemia and acute myeloid leukaemia: Support for histone deacetylase inhibitors as antileukaemic agents. Br. J. Haematol..

[73] Mummery A., Narendran A., Lee K.Y. (2011). Targeting epigenetics through histone deacetylase inhibitors in acute lymphoblastic leukemia. Curr. Cancer Drug Targets.

[74] Vilas-Zornoza A., Agirre X., Abizanda G., Moreno C., Segura V., De Martino Rodriguez A., José-Eneriz E.S., Miranda E., Martín-Subero J.I., Garate L. (2012). Preclinical activity of LBH589 alone or in combination with chemotherapy in a xenogeneic mouse model of human acute lymphoblastic leukemia. Leukemia.

[75] Janczar K., Janczar S., Pastorczak A., Mycko K., Paige A.J., Zalewska-Szewczyk B., Wagrowska-Danilewicz M., Danilewicz M., Mlynarski W. (2015). Preserved global histone H4 acetylation linked to ETV6-RUNX1 fusion and PAX5 deletions is associated with favorable outcome in pediatric B-cell progenitor acute lymphoblastic leukemia. Leuk. Res..

[76] Advani A.S., Gibson S.E., Douglas E., Jin T., Zhao X., Kalaycio M., Copelan E., Sobecks R., Sekeres M., Sungren S., Hsi E.D. (2010). Histone H4 acetylation by immunohistochemistry and prognosis in newly diagnosed adult acute lymphoblastic leukemia (ALL) patients. BMC Cancer.

[77] Advani A.S., Gibson S., Douglas E., Diacovo J., Elson P., Kalaycio M., Copelan E., Sekeres M., Sobecks R., Sungren S. (2011). Histone H4 acetylation by immunohistochemistry and prognosis in relapsed acute lymphocytic leukaemia (ALL). Br. J. Haematol..

[78] Bachmann P.S., Piazza R.G., Janes M.E., Wong N.C., Davies C., Mogavero A., Bhadri V.A., Szymanska B., Geninson G., Magistroni V. (2010). Epigenetic silencing of BIM in glucocorticoid poor-responsive pediatric acute lymphoblastic leukemia, and its reversal by histone deacetylase inhibition. Blood.

[79] Vu L.P., Luciani L., Nimer S.D. (2013). Histone-modifying enzymes: Their role in the pathogenesis of acute leukemia and their therapeutic potential. Int. J. Hematol..

[80] Chen C.W., Armstrong S.A. (2015). Targeting DOT1L and HOX gene expression in MLL-rearranged leukemia and beyond. Exp. Hematol..

[81] Bernt K.M., Armstrong S.A. (2011). Targeting epigenetic programs in MLL-rearranged leukemias. Hematol. Am. Soc. Hematol. Educ. Program.

[82] Mullighan C.G. (2012). Molecular genetics of B-precursor acute lymphoblastic leukemia. J. Clin. Investig..

[83] Rao R.C., Dou Y. (2015). Hijacked in cancer: The KMT2 (MLL) family of methyltransferases. Nat. Rev. Cancer.

[84] Bernt K.M., Zhu N., Sinha A.U., Vempati S., Faber J., Krivtsov A.V., Feng Z., Punt N., Daigle A., Bullinger L. (2011). MLL-rearranged leukemia is dependent on aberrant H3K79 methylation by DOT1L. Cancer Cell.

[85] Daigle S.R., Olhava E.J., Therkelsen C.A., Majer C.R., Sneeringer C.J., Song J., Johnston L.D., Scott M.P., Smith J.J., Xiao Y. (2011). Selective killing of mixed lineage leukemia cells by a potent small-molecule DOT1L inhibitor. Cancer Cell.

[86] Daigle S.R., Olhava E.J., Therkelsen C.A., Basavapathruni A., Jin L., Boriack-Sjodin P.A., Allain C.J., Klaus C.R., Raimondi A., Scott M.P. (2013). Potent inhibition of DOT1L as treatment of MLL-fusion leukemia. Blood.

[87] Deshpande A.J., Chen L., Fazio M., Sinha A.U., Bernt K.M., Banka D., Dias S., Chang J., Olhava E.J., Daigle S.R. (2013). Leukemic transformation by the MLL-AF6 fusion oncogene requires the H3K79 methyltransferase Dot1l. Blood.

[88] Bernt K.M., Armstrong S.A. (2011). A role for DOT1L in MLL-rearranged leukemias. Epigenomics.

[89] Ge Z., Song E.J., Kawasawa Y.I., Li J., Dovat S., Song C. (2016). WDR5 high expression and its effect on tumorigenesis in leukemia. Oncotarget.

[90] Cao F., Townsend E.C., Karatas H., Xu J., Li L., Lee S., Liu L., Chen Y., Ouillette P., Zhu J. (2014). Targeting MLL1 H3K4 methyltransferase activity in mixed-lineage leukemia. Mol. Cell.

[91] Jaffe J.D., Wang Y., Chan H.M., Zhang J., Huether R., Kryukov G.V., Bhang H.E., Taylor J.E., Hu M., Englund N.P. (2013). Global chromatin profiling reveals NSD2 mutations in pediatric acute lymphoblastic leukemia. Nat. Genet..

[92] Oyer J.A., Huang X., Zheng Y., Shim J., Ezponda T., Carpenter Z., Allegretta M., Okot-Kotber C.I., Patel J.P., Melnick A. (2014). Point mutation E1099K in MMSET/NSD2 enhances its methyltranferase activity and leads to altered global chromatin methylation in lymphoid malignancies. Leukemia.

[93] Zhu X., He F., Zeng H., Ling S., Chen A., Wang Y., Yan X., Wei W., Pang Y., Cheng H. (2014). Identification of functional cooperative mutations of SETD2 in human acute leukemia. Nat. Genet..

[94] Schäfer V., Ernst J., Rinke J., Winkelmann N., Beck J.F., Hochhaus A., Gruhn B., Ernst T. (2016). EZH2 mutations and promoter hypermethylation in childhood acute lymphoblastic leukemia. J. Cancer Res. Clin. Oncol..

[95] Mullighan C.G., Su X., Zhang J., Radtke I., Phillips L.A., Miller C.B., Ma J., Liu W., Cheng C., Schulman B.A. (2009). Deletion of IKZF1 and prognosis in acute lymphoblastic leukemia. N. Engl. J. Med..

[96] Van der Meulen J., Sanghvi V., Mavrakis K., Durinck K., Fang F., Matthijssens F., Rondou P., Rosen M., Pieters T., Vandenberghe P. (2015). The H3K27me3 demethylase UTX is a gender-specific tumor suppressor in T-cell acute lymphoblastic leukemia. Blood.

[97] Wang H., Song C., Ding Y., Pan X., Ge Z., Tan B.H., Gowda C., Sachdev M., Muthusami S., Ouyang H. (2016). Transcriptional Regulation of JARID1B/KDM5B Histone Demethylase by Ikaros, Histone Deacetylase 1 (HDAC1), and Casein Kinase 2 (CK2) in B-cell Acute Lymphoblastic Leukemia. J. Biol. Chem..

[98] Song C., Gowda C., Pan X., Ding Y., Tong Y., Tan B.H., Wang H., Muthusami S., Ge Z., Sachdev M. (2015). Targeting casein kinase II restores Ikaros tumor suppressor activity and demonstrates therapeutic efficacy in high-risk leukemia. Blood.

[99] Mullighan C.G., Zhang J., Harvey R.C., Collins-Underwood J.R., Schulman B.A., Phillips L.A., Tasian S.K., Loh M.L., Su X., Liu W. (2009). JAK mutations in high-risk childhood acute lymphoblastic leukemia. Proc. Natl. Acad. Sci. USA.

[100] Roberts K.G., Li Y., Payne-Turner D., Harvey R.C., Yang Y.L., Pei D., McCastlain K., Ding L., Lu C., Song G. (2014). Targetable kinase-activating lesions in Ph-like acute lymphoblastic leukemia. N. Engl. J. Med..

[101] Dawson M.A., Bannister A.J., Göttgens B., Foster S.D., Bartke T., Green A.R., Kouzarides T. (2009). JAK2 phosphorylates histone H3Y41 and excludes HP1alpha from chromatin. Nature.

[102] Maude S.L., Tasian S.K., Vincent T., Hall J.W., Sheen C., Roberts K.G., Seif A.E., Barrett D.M., Chen I.M., Collins J.R. (2012). Targeting JAK1/2 and mTOR in murine xenograft models of Ph-like acute lymphoblastic leukemia. Blood.

[103] Kucine N., Marubayashi S., Bhagwat N., Papalexi E., Koppikar P., Sanchez Martin M., Dong L., Tallman M.S., Paietta E., Wang K. (2015). Tumor-specific HSP90 inhibition as a therapeutic approach in JAK-mutant acute lymphoblastic leukemias. Blood.

[104] Loudin M.G., Wang J., Leung H.C., Gurusiddappa S., Meyer J., Condos G., Morrison D., Tsimelzon A., Devidas M., Heerema N.A. (2011). Genomic profiling in Down syndrome acute lymphoblastic leukemia identifies histone gene deletions associated with altered methylation profiles. Leukemia.

[105] Holmfeldt L., Wei L., Diaz-Flores E., Walsh M., Zhang J., Ding L., Payne-Turner D., Churchman M., Andersson A., Chen S.C. (2013). The genomic landscape of hypodiploid acute lymphoblastic leukemia. Nat. Genet..

[106] Cedar H., Bergman Y. (2011). Epigenetics of haematopoietic cell development. Nat. Rev. Immunol..

[107] Lotem J., Sachs L. (2006). Epigenetics and the plasticity of differentiation in normal and cancer stem cells. Oncogene.

[108] Feinberg A.P., Koldobskiy M.A., Göndör A. (2016). Epigenetic modulators, modifiers and mediators in cancer aetiology and progression. Nat. Rev. Genet..

[109] Haery L., Thompson R.C., Gilmore T.D. (2015). Histone acetyltransferases and histone deacetylases in B- and T-cell development, physiology and malignancy. Genes Cancer.

[110] Johnson K., Shapiro-Shelef M., Tunyaplin C., Calame K. (2005). Regulatory events in early and late B-cell differentiation. Mol. Immunol..

[111] Liu Y., Subrahmanyam R., Chakraborty T., Sen R., Desiderio S. (2007). A plant homeodomain in RAG-2 that binds Hypermethylated lysine 4 of histone H3 is necessary for efficient antigen-receptor-gene rearrangement. Immunity.

[112] Matthews A.G., Kuo A.J., Ramón-Maiques S., Han S., Champagne K.S., Ivanov D., Gallardo M., Carney D., Cheung P., Ciccone D.N. (2007). RAG2 PHD finger couples histone H3 lysine 4 trimethylation with V(D)J recombination. Nature.

[113] Ramón-Maiques S., Kuo A.J., Carney D., Matthews A.G., Oettinger M.A., Gozani O., Yang W. (2007). The plant homeodomain finger of RAG2 recognizes histone H3 methylated at both lysine-4 and arginine-2. Proc. Natl. Acad. Sci. USA.

[114] Shimazaki N., Tsai A.G., Lieber M.R. (2009). H3K4me3 stimulates the V(D)J RAG complex for both nicking and hairpinning in trans in addition to tethering in CIS: Implications for translocations. Mol. Cell.

[115] Shimazaki N., Lieber M.R. (2014). Histone methylation and V(D)J recombination. Int. J. Hematol..

[116] Johnson K., Angelin-Duclos C., Park S., Calame K.L. (2003). Changes in histone acetylation are associated with differences in accessibility of V(H) gene segments to V-DJ recombination during B-cell ontogeny and development. Mol. Cell. Biol..

[117] Espinoza C.R., Feeney A.J. (2005). The extent of histone acetylation correlates with the differential rearrangement frequency of individual VH genes in pro-B cells. J. Immunol..

[118] Nightingale K.P., Baumann M., Eberharter A., Mamais A., Becker P.B., Boyes J. (2007). Acetylation increases access of remodelling complexes to their nucleosome targets to enhance initiation of V(D)J recombination. Nucleic Acids Res..

[119] Papaemmanuil E., Rapado I., Li Y., Potter N.E., Wedge D.C., Tubio J., Alexandrov L.B., Van Loo P., Cooke S.L., Marshall J. (2014). RAG-mediated recombination is the predominant driver of oncogenic rearrangement in ETV6-RUNX1 acute lymphoblastic leukemia. Nat. Genet..

[120] Dong Y., Liu F., Wu C., Li S., Zhao X., Zhang P., Jiao J., Yu X., Ji Y., Zhang M. (2016). Illegitimate RAG-mediated recombination events are involved in IKZF1 Δ3–6 deletion in BCR-ABL1 lymphoblastic leukaemia. Clin. Exp. Immunol..

[121] Heinäniemi M., Vuorenmaa T., Teppo S., Kaikkonen M.U., Bouvy-Liivrand M., Mehtonen J., Niskanen H., Zachariadis V., Laukkanen S., Liuksiala T. (2016). Transcription-coupled genetic instability marks acute lymphoblastic leukemia structural variation hotspots. eLife.

[122] Figueroa M.E., Chen S.C., Andersson A.K., Phillips L.A., Li Y., Sotzen J., Kundu M., Downing J.R., Melnick A., Mullighan C.G. (2013). Integrated genetic and epigenetic analysis of childhood acute lymphoblastic leukemia. J. Clin. Investig..

[123] Mullighan C.G. (2013). Genomic characterization of childhood acute lymphoblastic leukemia. Semin. Hematol..

[124] Wang Q., Cheng T. (2014). Evidences for mutations in the histone modifying gene SETD2 as critical drivers in leukemia development. Sci. China Life Sci..

[125] Harvey R.C., Mullighan C.G., Chen I.M., Wharton W., Mikhail F.M., Carroll A.J., Kang H., Liu W., Dobbin K.K., Smith M.A. (2010). Rearrangement of CRLF2 is associated with mutation of JAK kinases, alteration of IKZF1, Hispanic/Latino ethnicity, and a poor outcome in pediatric B-progenitor acute lymphoblastic leukemia. Blood.

[126] Loh M.L., Zhang J., Harvey R.C., Roberts K., Payne-Turner D., Kang H., Wu G., Chen X., Becksfort J., Edmonson M. (2013). Tyrosine kinome sequencing of pediatric acute lymphoblastic leukemia: A report from the Children’s Oncology Group TARGET Project. Blood.

